# Central Venous-To-Arterial CO_2_-Gap May Increase in Severe Isovolemic Anemia

**DOI:** 10.1371/journal.pone.0105148

**Published:** 2014-08-19

**Authors:** Szilvia Kocsi, Gábor Demeter, Dániel Érces, József Kaszaki, Zsolt Molnár

**Affiliations:** 1 Department of Anaesthesiology and Intensive Therapy, University of Szeged, Szeged, Hungary; 2 Department of Anaesthesiology and Intensive Therapy, Hungarian Defence Forces Military Hospital, Budapest, Hungary; 3 Institute of Surgical Research, University of Szeged, Szeged, Hungary; University of Louisville, United States of America

## Abstract

Despite blood transfusions are administered to restore adequate tissue oxygenation, transfusion guidelines consider only hemoglobin as trigger value, which gives little information about the balance between oxygen delivery and consumption. Central venous oxygen saturation is an alternative, however its changes reflect systemic metabolism and fail to detect regional hypoxia. A complementary parameter to ScvO_2_ may be central venous-to-arterial carbon dioxide difference (CO_2_-gap). Our aim was to investigate the change of alternative transfusion trigger values in experimental isovolemic anemia. After splenectomy, anesthetized Vietnamese mini pigs (n = 13, weight range: 18–30 kg) underwent controlled bleeding in five stages (T_1_–T_5_). During each stage approximately 10% of the estimated starting total blood volume was removed and immediately replaced with an equal volume of colloid. Hemodynamic measurements and blood gas analysis were then performed. Each stage of bleeding resulted in a significant fall in hemoglobin, the O_2_-extraction increased significantly from T_3_ and ScvO_2_ showed a similar pattern and dropped below the physiological threshold of 70% at T_4_. By T_4_ CO_2_-gap increased significantly and well correlated with VO_2_/DO_2_ and ScvO_2_. To our knowledge, this is the first study to show that anemia caused altered oxygen extraction may have an effect on CO_2_-gap.

## Introduction

Transfusion of red blood cells is an everyday practice in critical care with the primary aim of restoring adequate tissue oxygenation. Transfusion guidelines consider certain levels of hemoglobin as transfusion trigger [1], [2], which on its own gives little information if any about the balance between oxygen delivery (DO_2_) and consumption (VO_2_). Hence, there is a clear need for additional physiologic transfusion trigger values. One of the potentially useful physiological parameters is the central venous oxygen saturation (ScvO_2_), which has been shown to be a potential physiologic transfusion trigger in hemodynamically stable but anemic patients [3]. Its normal value is around 70–75% and it is the product of the VO_2_ and DO_2_ relationship. Low ScvO_2_ usually indicates inadequate DO_2_, but higher than physiological values may be difficult to interpret as these can indicate reduced oxygen consumption, but may also mean inappropriate oxygen uptake [4], [5]. Under these circumstances additional parameters are needed.

Central venous-to-arterial carbon dioxide difference (CO_2_-gap) may be one of the potential alternatives to complement ScvO_2_. Under physiological circumstances its value is less than 6 mmHg [6], [7]. Transport of carbon dioxide in blood ensues in three forms: dissolved in plasma, as bicarbonate ion and bound to hemoglobin. The CO_2_-gap may be higher during anaerobic respiration when lactic acid has to be buffered by bicarbonate or under aerobic respiration in poorly perfused tissues when flow stagnation results in an accumulation of CO_2_ [8], [9], [10]. From previous experiments it seems that increased CO_2_-gap during ischemia is related to decreased blood flow and impaired CO_2_ washout rather than to hypoxemia [10]. Whether anemia caused tissue hypoxemia is reflected in changes of the CO_2_-gap has not been investigated before.

Another additional parameter may be the central venous-to-arterial pCO_2_ difference divided by the difference of the arterio-venous oxygen content, P(v-a)CO_2_/C(a-v)O_2_, which is considered to give information about tissue oxygenation. It was found in a retrospective study that this ratio reflected the occurrence of anaerobic metabolism better than other oxygen-, or CO_2_-derived parameters [11].

Our aim was to investigate how CO_2_-gap and P(v-a)CO_2_/C(a-v)O_2_ change during experimental isovolemic anemia.

## Materials and Methods

The study protocol was approved by the local ethics committee at the University of Szeged and the study was carried out in the research laboratory of the Institute of Surgical Research. The current experiment complements our previously published data on the relationship of ScvO_2_ and isovolemic anemia [12]. Vietnamese mini pigs (n = 13) weighing 24±3 kg were anaesthetized and mechanically ventilated in pressure control mode. Anesthesia was induced with an intramuscular injection of a mixture of ketamine (20 mg/kg) and xylazine (2 mg/kg) and maintained with a continuous infusion of propofol (6 mg/kg/h i.v.). The tidal volume was set at 13±2 ml/kg and the respiratory rate was adjusted to maintain the end-tidal carbon dioxide and the partial pressure of arterial carbon dioxide in the range of 35–45 mmHg and the arterial pH between 7.35 and 7.45. The adequacy of the depth of anesthesia was assessed by monitoring the jaw tone. After the initiation of anesthesia, the right carotid artery and jugular vein and the right femoral artery and vein were dissected and catheterized. The animals underwent suprapubic urinary catheter placement and laparotomy for splenectomy. Splenectomy in swine hemorrhage models are performed because of the distensibility of the spleen and the resultant variation in the amounts of sequestered blood [13]. The core temperature was maintained at 37±1°C through use of an external warming device.

For invasive hemodynamic monitoring, a transpulmonary thermodilution catheter (PiCCO, PULSION Medical Systems AG, Munich, Germany) was placed in the femoral artery and a pulmonary artery catheter (PV2057 VoLEF Catheter, PULSION Medical Systems AG) by pressure tracings via the femoral vein. The latter was also used to draw mixed venous blood gas samples. The femoral artery served as the site of arterial blood gas samples and the central venous line was used for central venous blood gas sampling and for the injection of cold saline boluses for thermodilution measurements. Central venous catheter was positioned by using guidewire attached intracavital ECG. During the experiment blood was drained from the catheter in the right carotid artery, which was also used to replace the blood loss with the same amount of colloid, in order to avoid a sudden increase in right ventricular preload.

At baseline (T_0_) hemodynamic and blood gas parameters were recorded, and heparin sulfate (200 IU/kg) was administered through the central venous line. Isovolemic anemia was achieved in five intervals (T_1_–T_5_). During each interval 10% of the estimated total blood volume was withdrawn over a 5- to 10-min period. Hemodynamic parameters were recorded and the amount of blood drained was immediately replaced by an equal volume of colloid (hydroxyethyl starch 130 kDa/0.4, 6%, Voluven, Fresenius, Germany). To achieve a steady state, the animals were allowed to rest for 10 min between intervals. At the end of each cycle, hemodynamic and blood gas parameters were measured. At the end of the experiment the animals were humanely euthanized.

Arterial, central venous, and mixed venous blood gas samples (Cobas b 221, Roche Ltd., Basel, Switzerland) were drawn and analyzed by cooximetry simultaneously at baseline and at the end of each cycle. From these parameters the oxygen delivery (DO_2_), oxygen consumption (VO_2_), oxygen extraction ratio (VO_2_/DO_2_) and the simplified oxygen extraction ratio (ERO_2_) were calculated according to standard formulae:
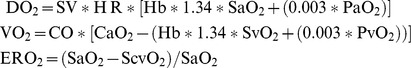



Central venous-to-arterial CO_2_-gap (_cv_CO_2_-gap), mixed venous-to-arterial CO_2_-gap (_v_CO_2_-gap), the P_(cv-a)_CO_2_/C_(a-cv)_O_2_ and P_(v-a)_CO_2_/C_(a-v)_O_2_ were also calculated from the arterial, central venous and mixed venous blood gas samples.

These were calculated according to standard formulae:
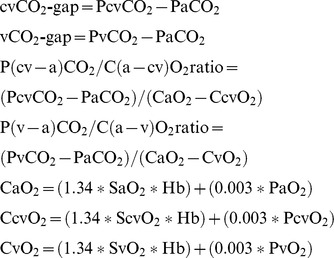



## Analysis

Data are reported as median±standard deviation unless indicated otherwise. For testing normal distribution the Kolmogorov-Smirnov test was used. Changes in all parameters throughout the experiment were tested by Friedman test and repeated measures analysis of variance (RM ANOVA), and the number of degrees of freedom was adjusted to Greenhouse-Geisser epsilon when needed. For pairwise comparisons Pearson's correlation was used. To evaluate the performance in detecting altered oxygen extraction of >30% (considered as the “physiological threshold”), receiver operating characteristics (ROC) curve analysis was performed. Post-hoc calculation showed a power of 86% with an effect of 25% increase in VO_2_/DO_2_, for a sample size of 13 and α = 0.05. For statistical analysis SPSS version 20.0 for Windows (SPSS, Chicago, IL, USA) was used and p<0.05 was considered statistically significant.

## Results

**Figure 1:**
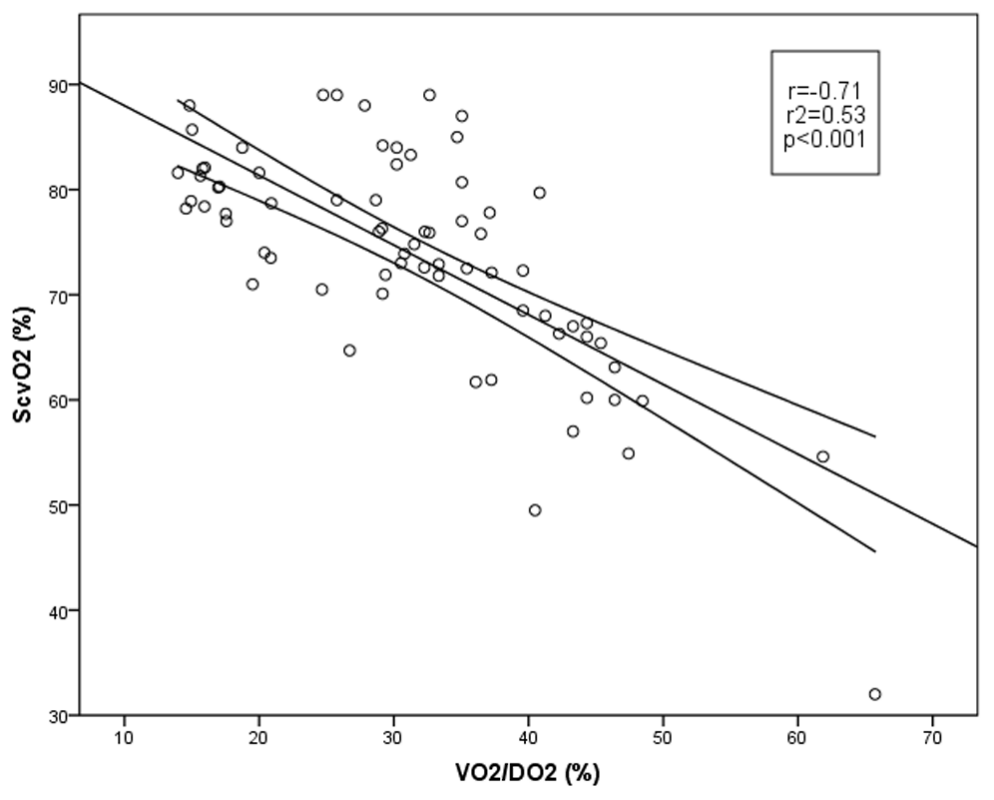
The association between VO_2_/DO_2_ and ScvO_2_. VO_2_/DO_2_: oxygen extraction ratio; ScvO_2_: central venous oxygen saturation.

All 13 animals survived the study. The bleeding caused a gradual decrease in hemoglobin level after each phase and by the end of the experiment it had fallen by 61% of the baseline value. The hemodynamic parameters are summarized in Table 1. The SaO_2_ remained in the normal range throughout the experiment. DO_2_ fell significantly from T_2_, VO_2_ at T_4_, VO_2_/DO_2_ increased significantly from T_3_, and exceeded the physiologic threshold of 30% (Table 2). The change in ScvO_2_ displayed a similar pattern as VO_2_/DO_2_ and changed significantly and also fell below 70% only at T_4_. There was strong negative correlation between VO_2_/DO_2_ and ScvO_2_ (Fig. 1).

**Table 1:** Hemodynamic effects of isovolemic anemia. These data have been published earlier [12].

	T_0_	T_1_	T_2_	T_3_	T_4_	T_5_
Hb (g/L)	125(113–134)	102(90–109)* ^#^	79(73–93)* ^#^	68(60–76)* ^#^	59(53–67)* ^#^	49(43–55)* ^#^
HR (beats/min)	125(91–135)	119(100–138)*	123(102–146)*	129(110–159) *	139(118–179) *	147(131–177)*
MAP (mm Hg)	91(79–105)	89(79–101)	83(75–98)*	82(68–90)*	72(59–85)*	72(63–86)*
CVP (mm Hg)	6(5–8)	8(5–9)	7(4–9)	7(5–9)	7(5–9)	7(3–10)
CI (L/min/m^2^)	2.6(2.3–2.8)	3.3(2.7–3.6)* ^#^	3.6(2.9–3.8)* ^#^	3.6(3.3–4.1)*	3.5(3.2–4.0)*	3.9(3.6–4.1)*
GEDI (mL/m^2^)	270 (243–284)	271 (245–320)	276 (248–298)	274 (236–305)	268 (227–302)	261 (232–298)
ELWI (mL/kg)	9 (9–10)	10 (10–10)	9 (9–10)	10 (9–10)	10 (9–10)	10 (9–11)
dPmx (mm Hg/s)	540(485–790)	700(540–985)*	800(570–1075)*	810(540–1480)*	880(560–1360)*	975(562–1275)*

Hb- Hemoglobin, HR- Heart rate, MAP- Mean arterial pressure, CVP- Central venous pressure, CI- Cardiac index, GEDI- Global end-diastolic volume index, ELWI- extravascular lung water index, dPmx- Index of left ventricular contractility. T_0_- Baseline measurement, T_1_-T_5_- Five intervals of bleeding.

*p<.05 compared with T_0_; ^#^p<.05 compared with previous; GLM repeated measures ANOVA.

**Table 2:** Descriptives (Median±IQR).

	Time intervals					
	T_0_	T_1_	T_2_	T_3_	T_4_	T_5_
_cv_CO_2_-gap (mmHg)	5.0(2.6–8.5)	6.0(3.1–7.0)	5.0(3.5–5.5)	5.4(4.4–7.0)	8.0(4.3–8.5)*	6.3(5.9–11.0)*
_v_CO_2_-gap (mmHg)	5.5(4.0–9.0)	6.5(4.5–7.8)	6.5(5.1–7.0)	5.5(3.7–6.0)	5.4(5.0–8.0)	6.2(5.5–8.0)
P_cv_CO_2_/C_(a-cv)_O_2_	2.01(1.42–2.23)	2.27(1.76–3.34)	2.67(1.71–2.85)	2.59(1.50–4.47)	3.30(2.89–3.74)*	3.93(2.55–5.11)*
P_v_CO_2_/C_(a-v)_O_2_	1.57(0.77–1.99)	1.69(0.91–2.00)	1.71(1.36–1.99)	1.61(0.96–2.17)	2.14(1.58–2.23)	2.30(1.93–3.56)*
ScvO_2_ (%)^#^	76(69–83)	73(72–82)	77(75–83)	77(68–81)	68(61–76)*	66(60–76)*
SvO_2_ (%)^#^	68 (64–77)	67 (64–77)	68 (63–79)	64 (58–76)	62 (55–72)*	58 (52–72)*
DO_2_ (ml/min/m^2^) ^#^	431 (362–474)	438 (323–524)	378 (302–412)*	344 (252–376)*	284 (236–333)*	247 (216–292)*
VO_2_ (ml/min/m^2^) ^#^	119 (82–139)	130 (77–151)	93 (66–136)	113 (67–141)	98 (72–120)*	105 (70–120)*
VO_2_/DO_2_ (%)^#^	29(18–33)	29(17–33)	29(18–32)	35(21–40)*	37(26–43)*	41(27–47)*
ERO_2_ (%)^#^	19(13–26)	19(14–24)	20(14–22)	21(16–28)	30(22–37)*	32(21–39)*
Lactate (mmol/L) ^#^	4.5 (3.2–5.3)	4.2 (3.0–5.1)	5.0 (3.2–6.0)	4.1 (2.9–6.0)	4.2 (2.9–6.5)	4.0 (3.0–6.4)
vLactate (mmol/L)	4.6(3.7–5.3)	4.3(3.3–5.3)	4.4(3.1–5.4)	4.4(2.8–5.2)	4.4(3.0–5.2)	4.1(3.0–6.4)
cvLactate (mmol/L)	4.5(3.5–5.5)^§^	3.9(3.4–5.4)^§^	4.2(3.3–6.3)^§^	4.1(3.1–5.6)^§^	3.9(2.9–5.7)^§^	3.9(3.0–6.4)^§^
PaCO_2_ (mmHg) ^#^	39(35–44)	38(35–45)	37(34–45)	39(34–46)	37(34–42)	38(35–41)
PaO_2_ (mmHg) ^#^	76(66–80)	75(72–80)	76(73–80)	77(72–82)	79(75–85)	81(77–90)

_cv_CO_2_-gap: central venous-to-arterial carbon dioxide difference; _v_CO_2_-gap: mixed venous-to-arterial carbon dioxide difference; P_(cv-a)_CO_2_/C_(a-cv)_O_2_: the central venous-to-arterial pCO_2_ difference divided by the difference of the arterio-venous oxygen content; P_(v-a)_CO_2_/C_(a-v)_O_2_: the mixed venous-to-arterial pCO_2_ difference divided by the difference of the arterio-venous oxygen content; ScvO_2_: central venous oxygen saturation; SvO_2_: mixed venous oxygen saturation; DO_2_: oxygen delivery; VO_2_: oxygen consumption; VO_2_/DO_2_: oxygen extraction ratio; ERO_2_: simplified oxygen extraction ratio; PaCO_2_: arterial partial pressure of carbon dioxide; PaO_2_: arterial partial pressure of oxygen * p<.05 as compared to baseline, ^§^ p<.05 significant difference between mixed venous and central venous blood with Friedman and Wilcoxon tests, ^#^ Data published earlier [12].

**Figure 2:**
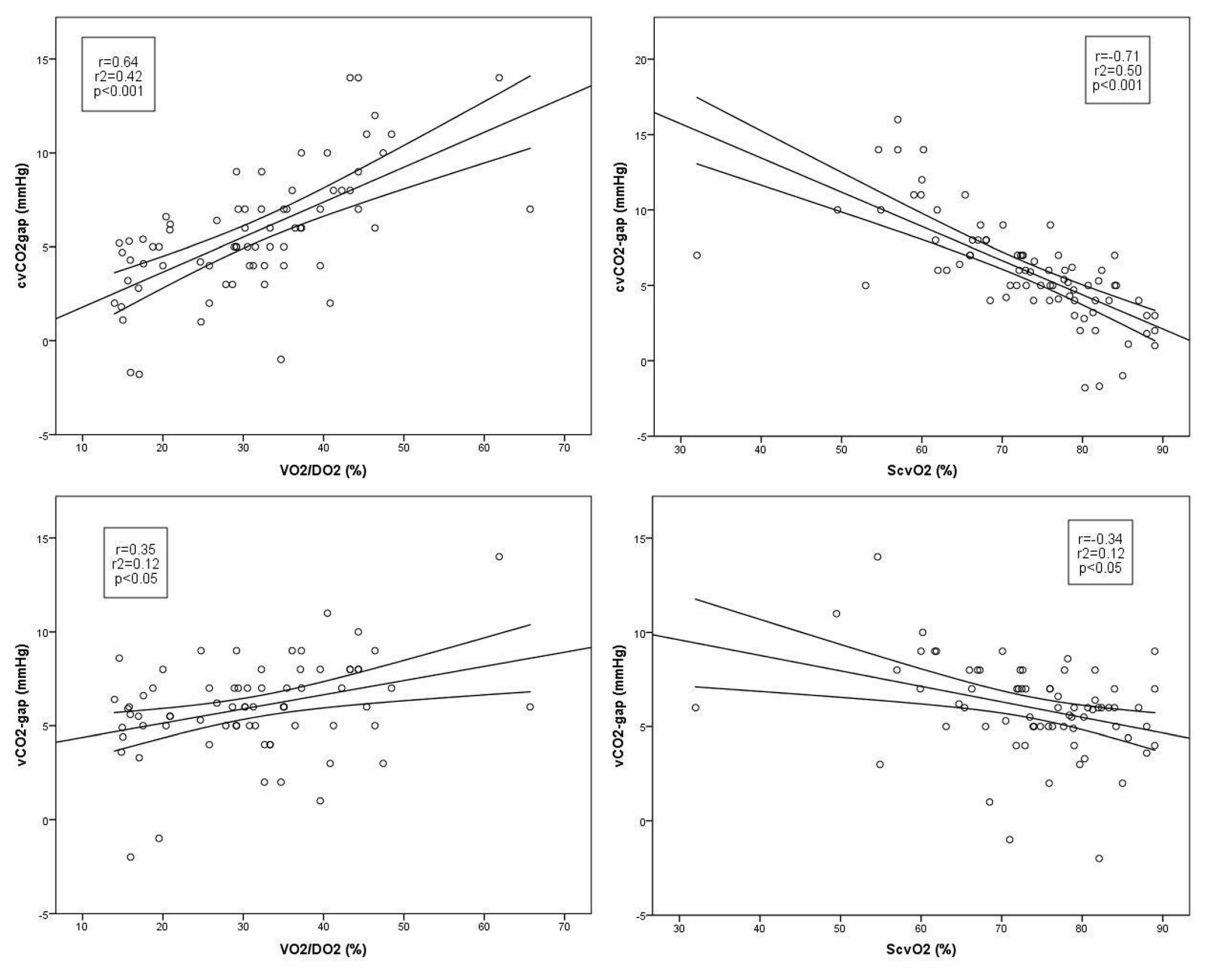
Correlation between oxygen balance parameters and CO_2_-gap. _cv_CO_2_-gap and VO_2_/DO_2_ and ScvO_2_ (on the left); _v_CO_2_-gap and VO_2_/DO_2_ and ScvO_2_ (on the right). cvCO_2_-gap: central venous-to-arterial carbon dioxide difference; VO_2_/DO_2_: oxygen extraction ratio; ScvO_2_: central venous oxygen saturation; vCO_2_-gap: mixed venous-to-arterial carbon dioxide difference.

The CO_2_-gap was calculated for both, central venous (_cv_CO_2_-gap) and mixed venous blood (_v_CO_2_-gap). By T_4__cv_CO_2_-gap increased significantly, however _v_CO_2_-gap did not change. The correlations of VO_2_/DO_2_ and ScvO_2_ were significant with _cv_CO_2_-gap, while there were only weak correlations with _v_CO_2_-gap (Fig. 2).

**Figure 3:**
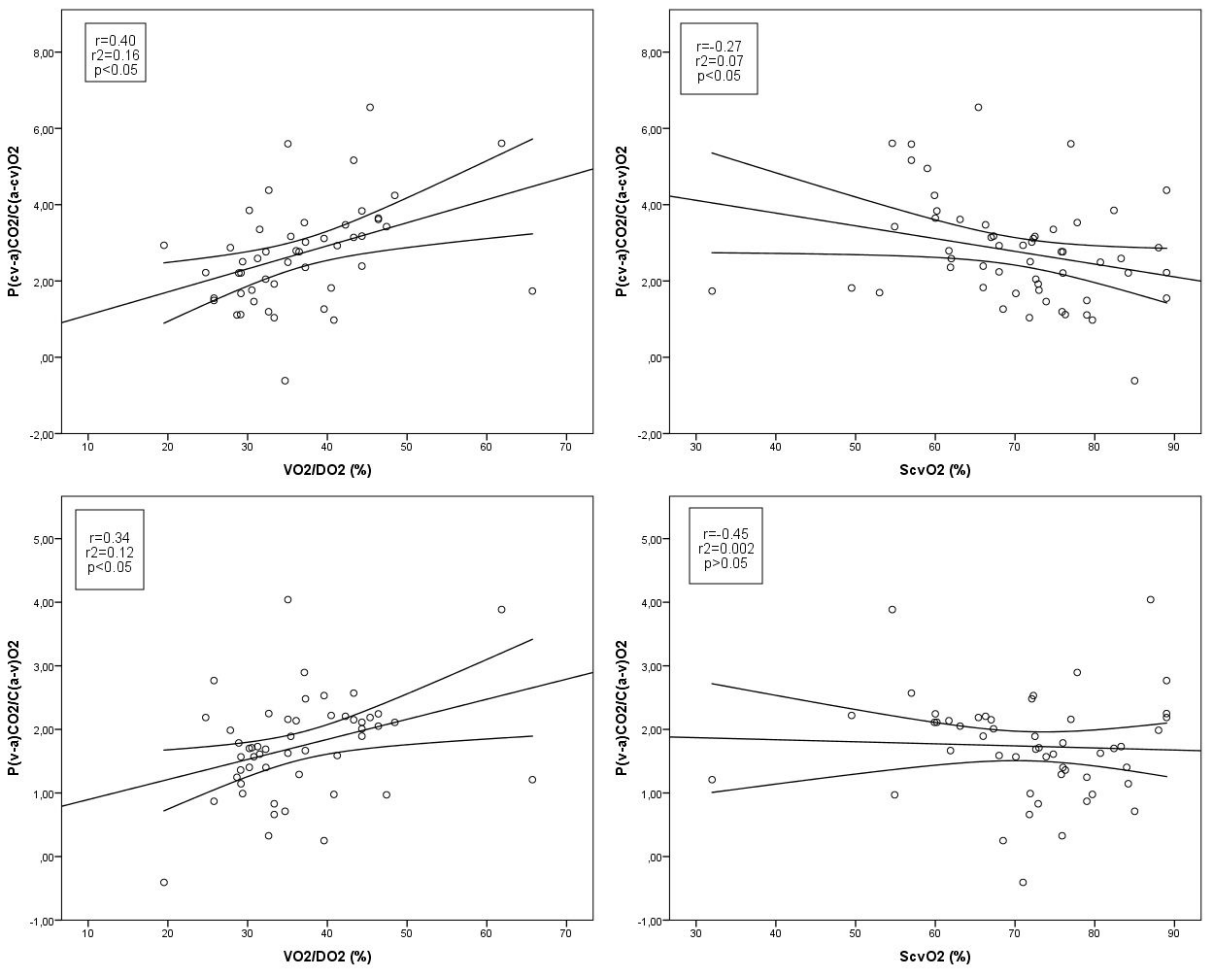
Correlation between tissue oxygenation and oxygen balance parameters. P_(cv-a)_CO_2_/C_(a-cv)_O_2_ and VO_2_/DO_2_ and ScvO_2_ (on the left); P_(v-a)_CO_2_/C_(a-v)_O_2_ and VO_2_/DO_2_ and ScvO_2_ (on the right).P_(cv-a)_CO_2_/C_(a-cv)_O_2_: the central venous-to-arterial pCO_2_ difference divided by the difference of the arterio-venous oxygen content; VO_2_/DO_2_: oxygen extraction ratio; ScvO_2_: central venous oxygen saturation; P_(v-a)_CO_2_/C_(a-v)_O_2_: the mixed venous-to-arterial pCO_2_ difference divided by the difference of the arterio-venous oxygen content.

P_(cv-a)_CO_2_/C_(a-cv)_O_2_ increased by T_4_ and P_(v-a)_CO_2_/C_(a-v)_O_2_ by T_5_. The correlations of VO_2_/DO_2_ and ScvO_2_ were significant with P_(cv-a)_CO_2_/C_(a-cv)_O_2_, but it was found to be weak between P_(v-a)_CO_2_/C_(a-v)_O_2_ and VO_2_/DO_2_, and there was no significant correlation with ScvO_2_ (Fig. 3).

ROC analysis revealed the same tendency as the correlation. With 30% taken as the physiologic threshold for VO_2_/DO_2_, the area under the curve (AUC), its standard error and that of the 95% confidence interval were >0.5 only for _cv_CO_2_-gap, P_(cv-a)_CO_2_/C_(a-cv)_O_2_ ratio, ScvO_2_ (Table 3.)

**Table 3:** ROC analysis for determining VO_2_/DO_2_>30%.

Test Result Variable(s)	Area	Std. Error	Sig.	95% CI	
_cv_CO_2_-gap	,769	,078	,007	,617	,921
_v_CO_2_-gap	,553	,097	,598	,363	,742
P_(cv-a)_CO_2_/C_(a-cv)_O_2_ ratio	,742	,070	,016	,604	,879
P_(v-a)_CO_2_/C_(a-v)_O_2_ ratio	,641	,096	,157	,453	,829
ScvO_2_	,768	,056	,000	,657	,879
SvO_2_	,986	,010	,000	,967	1,000
Lactate	,517	,078	,867	,363	,670

_cv_CO_2_-gap: central venous-to-arterial carbon dioxide difference;

_v_CO_2_-gap: mixed venous-to-arterial carbon dioxide difference;

P_(cv-a)_CO_2_/C_(a-cv)_O_2_: the central venous-to-arterial pCO_2_ difference divided by the difference of the arterio-venous oxygen content;

P_(v-a)_CO_2_/C_(a-v)_O_2_: the mixed venous-to-arterial pCO_2_ difference divided by the difference of the arterio-venous oxygen content;

ScvO_2_: central venous oxygen saturation; SvO_2_: mixed venous oxygen saturation.

Linear regression revealed a significant relationship between ScvO_2_ (r = 0.71, r^2^ = 0.50, p<.001) and VO_2_/DO_2_. This relationship became significantly stronger when _cv_CO_2_-gap was added to ScvO_2_ (r = 0.74, r^2^ = 0.54, p = .015). According to the Pratt's importance coefficient, ScvO_2_ was responsible for this increase in 63% and _cv_CO_2_-gap in 37%.

## Discussion

Our results in this isovolemic anemia animal model show that besides ScvO_2_, both central venous-to-arterial CO_2_-gap and the P_(cv-a)_CO_2_/C_(a-cv)_O_2_ correlated well with changes in anemia caused increase in VO_2_/DO_2_. Furthermore, mixed venous blood driven indices, such as _v_CO_2_-gap and P_(v-a)_CO_2_/C_(a-v)_O_2_ failed to indicate changes in oxygen extraction. When oxygen extraction ratio started to increase (from T_3_) it was followed by a decrease of ScvO_2_ and an increase of _cv_CO_2_-gap and P_(cv-a)_CO_2_/C_(a-cv)_O_2_, and both performed well in the ROC analysis, with the _cv_CO_2_-gap's AUC being marginally better. In addition, in our experiment neither _v_CO_2_-gap nor P_(v-a)_CO_2_/C_(a-v)_O_2_ or lactate could detect the increase in VO_2_/DO_2_>30% as revealed by ROC analysis.

An interesting finding of our experiment is that despite isovolemia was maintained as indicated by the stable global end diastolic volume index values and there were in fact increasing cardiac output and stroke volume, we observed a rise in _cv_CO_2_-gap. This observation seemingly contradicts previously published results to some extent. The occurrence of increased CO_2_-gap has fundamentally been explained by the CO_2_ stagnation phenomenon [5]. This was based on the finding that there was inverse correlation between CO_2_-gap and cardiac index during non-septic and septic low flow states [5], [9], [10]. Moreover, it was also found that the amount of CO_2_ produced is negligible when anaerobic respiration is present and CO_2_-gap therefore cannot serve as a marker of tissue hypoxia [10]. The paramount study on this theory by Vallet et al. used an isolated hind limb model and reached hypoxia either by decreasing flow or decreasing arterial oxygen content [10]. They found that occurrence of an increased CO_2_-gap during ischemia was related to decreased blood flow and impaired carbon dioxide washout; moreover, dysoxia *per se* was not sufficient to increase CO_2_-gap. However, the latter could also be due to the Haldane's effect. As the carbon dioxide dissociation curve is influenced by the saturation of hemoglobin with oxygen, the lower the saturation of hemoglobin with oxygen, the higher the saturation of hemoglobin with carbon dioxide for a given carbon dioxide partial pressure is [14]. In our experiment arterial oxygen saturation and PaO_2_ remained in the normal range and did not change over time, hence the CO_2_ dissociation curve was not influenced by low saturation of hemoglobin with oxygen.

Nevertheless, anemia resulted in increased VO_2_/DO_2_ above the baseline and also above the physiological 30% after the 3^rd^ bleeding event, which was followed by the significant decrease of SvO_2_ and ScvO_2_. (It is important to note that there is mathematical coupling between VO_2_ and SvO_2_, which is not the case considering ScvO_2_). The most interesting finding of the current study is the increase of _cv_CO_2_-gap during the last two stages of the experiment, without any change in the _v_CO_2_-gap. One of the possible reasons for this difference is that due to isovolemia cardiac output was maintained to avoid low flow in the systemic circulation, which is also reinforced by the unchanged lactate levels. Therefore when CO_2_ was measured in the mixed venous blood it was unchanged and within the normal range almost throughout. As central venous blood driven variables mostly reflect blood flow and metabolism of the brain [15], our hypothesis is that anemia reached such a degree by T_4_ that it caused tissue hypoxia and consecutive anaerobic respiration with CO_2_ production. However, due to the low hemoglobin levels the Haldane effect could not take effect, hence there was a significant increase in central venous pCO_2_. But these changes in the brain did not have significant effects on the systemic level, to be picked up in mixed venous blood. As anemia has greater influence on arterial oxygenation than hypoxemia [16], this might explain the observed increase in _cv_CO_2_-gap. This is further reinforced by the P_(cv-a)_CO_2_/C_(a-cv)_O_2_ results. Both the P_(cv-a)_CO_2_/C_(a-cv)_O_2_ and the P_(v-a)_CO_2_/C_(a-v)_O_2_ increased at T_4_ and T_5_, but there was a more pronounced change in central venous as compared to mixed venous blood, which is also reflected in the results of the ROC analysis. We also measured mixed venous and central venous lactate levels and found that central venous lactate was significantly lower than in the mixed venous blood, which might give further proof to this hypothesis [17], [18]. In a previous animal experiment by Hare *et al*, it was found that hemodilutional isovolemic anemia led to cerebral hypoxia, and they also reported a gradual increase in the jugular venous pCO_2_ with a CO_2_-gap of 2.9 to 7.8 mmHg (mean) 60 minutes after hemodilution in the traumatic brain injured animals [19]. Although this finding was not discussed in the article, as the authors mainly focused on oxygenation, but nevertheless this is in accord with our results and gives some support to our hypothesis.

There is increasing evidence that untreated anemia can be associated with a worse outcome and increased mortality, while transfusion may cause various infectious and non-infectious adverse effects [20], [21]. _cv_CO_2_-gap may be an additional quantitative parameter, beyond Hb and ScvO_2_, that would give information on anemia related altered oxygen extraction and hence the need for blood administration. _cv_CO_2_-gap is a choice of plausible alternatives as it can be easily obtained via the central venous and arterial catheters already *in situ* in most critically ill patients and no additional invasive device is needed; moreover we found that mixed venous blood driven indices failed to indicate changes in oxygen extraction.

There are several limitations of our study. As the experiment was not designed to measure the effects of isovolemic anemia specifically on the brain, our hypothesis cannot be supported by specific measurements, such as regional cerebral blood flow, cerebral tissue oxygen and carbon dioxide tension. Furthermore, splenectomy and the length of the preparation of the animals may have been too long, which resulted in increased levels of lactate from baseline to the end of the experiment. The steady-state periods may also have been relatively short, although, the same time intervals have been used previously [22]. Another concern might be the type of fluid replacement, as one cannot exclude the possibility that the use of different types of colloid or crystalloid solutions would affect these results.

## Conclusions

To our knowledge, this is the first study to show that anemia caused altered oxygen extraction may have an effect on _cv_CO_2_-gap and P_(cv-a)_CO_2_/C_(a-cv)_O_2_ that cannot be detected from mixed venous blood. The clinical relevance of this finding has to be further tested in both experimental and clinical studies.
